# Thiopurines in the Management of Crohn's Disease: Safety and Efficacy Profile in Patients with Normal TPMT Activity—A Retrospective Study

**DOI:** 10.1155/2016/1034834

**Published:** 2016-03-29

**Authors:** Amine Benmassaoud, Xuanqian Xie, Motaz AlYafi, Yves Theoret, Alain Bitton, Waqqas Afif, Talat Bessissow

**Affiliations:** ^1^Division of Gastroenterology, McGill University Health Center, 1650 Cedar Avenue, C7-200, Montreal, QC, Canada H3G 1A4; ^2^Technology Assessment Unit, McGill University Health Center, 687 Pine Avenue West, Room R4.09, Montreal, QC, Canada H3A 1A1; ^3^Unite de Pharmacologie Clinique, Centre Hospitalier Universitaire de Sainte-Justine, 3175 Chemin de la Côte-Sainte-Catherine, Montreal, QC, Canada H3T 1C4

## Abstract

*Background and Aims.* Thiopurines are used in the treatment of Crohn's disease (CD) and thiopurine S-methyltransferase (TPMT) activity can guide thiopurine dosing to avoid adverse events. This retrospective study evaluated the safety and efficacy of starting thiopurines at low dose versus full dose in patients with CD and normal TPMT.* Methods.* This was a single center retrospective study including adult CD patients with normal TPMT levels (≥25 nmol/hr/g Hgb) who were followed for 1 year. Patients started at full dose of azathioprine (2–2.5 mg/kg) or 6-mercaptopurine (1–1.5 mg/kg) were compared to patients started at low dose. Harvey-Bradshaw index, treatment failure, and drug-related adverse events were recorded.* Results.* Our study included 134 patients. Both groups had similar incidences of drug-related adverse events and discontinuation of therapy due to side effects. Fifty-six percent of all adverse events occurred within 31 days and 92% occurred within 3 months of therapy. Clinical response favored the full-dose group at 6 months (69% versus 27%, *p* = 0.0542).* Conclusions.* Our study indicates that it is safe to start patients on full-dose thiopurine when they have a normal TPMT given its very similar toxicity profile to patients started on low dose. This may also positively impact efficacy.

## 1. Introduction

6-Mercaptopurine (6-MP) and its prodrug, azathioprine (AZA), have been used in the management of Crohn's disease (CD) for more than half a century [1]. Once absorbed, AZA and 6-MP undergo extensive modifications before they are converted into active metabolites, including 6-thioguanine nucleotides (6-TGN) [2]. Four to five weeks is required before the active metabolites reach steady state and anti-inflammatory effects are seen [3].

The most common side effects associated with thiopurine use include gastrointestinal intolerance, myelosuppression, hepatitis, pancreatitis, and flu-like syndromes [4–7]. The metabolism of thiopurines is dependent on a major enzymatic pathway that involves thiopurine S-methyltransferase (TPMT). TPMT activity reference intervals have been established using a large prospective study with 1000 individuals and normal activity was associated with a level equal to or above 25 nmol/hr/g Hgb [8]. In a population-based study, one in 300 patients has low to absent TPMT activity (homozygous mutant TPMT), 11% have intermediate TPMT activity (heterozygous TPMT), and 89% have normal to elevated TPMT activity (homozygous wild type TPMT) [9].

A meta-analysis of 5 studies looking at the association between hepatotoxicity and TPMT genotypes concluded that there was no association between the two [10]. However, deficiency in TPMT has been implicated in the occurrence of myelosuppression [11–13] and one meta-analysis of 9 studies demonstrated a positive association between heterozygous TPMT genotype and bone marrow suppression with an odds ratio (OR) of 5.93 (95% CI 2.96–11.88; *p* < 0.00001) [10]. The USA Food and Drug Administration (FDA) therefore recommends that clinicians measure TPMT activity levels (referred to as TPMT phenotype) or determine TPMT genotype before initiating thiopurine therapy to avoid myelotoxicity. The American College of Gastroenterology (ACG) also endorses this recommendation [14]. It is thought that the use of TPMT activity and 6-TGN level measurements could help avoid nearly a quarter of episodes of myelosuppression.

The Clinical Pharmacogenetics Implementation Consortium from the US National Institutes of Health provides recommendations on the optimal dosing strategy for thiopurines depending on TPMT activity. In patients with a normal to high TPMT activity level, AZA or 6-MP can be started at the target dose of 2.0–2.5 mg/kg per day or 1.0 mg/kg–1.5 mg/kg per day, respectively. Individuals with intermediate TPMT activity should start with a dose reduction of 30–70% given daily. Patients with low TPMT activity should be offered alternative therapy or started at 10% of the suggested dosing, given three times per week [15].

Despite these recommendations, physician prescriptions of thiopurines are not uniform and many patients are started on low-dose thiopurines despite documentation of a normal TPMT level, most likely due to a fear of major side effects [16–18]. This reticence may also result in failure to optimize or increase doses in patients with active disease. Physicians may also be reluctant to start patients on full-dose thiopurines because of the inability to ensure close monitoring of blood work.

Given these prescribing inconsistencies, we undertook this study to compare the safety and efficacy of thiopurines when initiated at full dose versus low dose, in CD patients with normal TPMT activity (≥25 nmol/hr/g Hgb) [8].

## 2. Materials and Methods

We conducted a retrospective single center study at the McGill University Health Center (MUHC) that included all CD patients started on AZA or 6-MP with a known TPMT activity ≥ 25 nmol/hr/g Hgb. Subjects were identified using a database of all patients with a measured TPMT level and a subsequent chart review was performed to identify those that were started on a thiopurine between 2008 and 2012. To be included in the study, subjects had to have been followed for a minimum of 12 months. Exclusion criteria included prior use of a thiopurine, initiation of a thiopurine before knowledge of TPMT level, thiopurine use for postoperative prophylaxis, blood transfusion within 3 months before TPMT activity determination, concurrent use of anti-TNF agents or allopurinol at initiation of thiopurine therapy, creatinine clearance less than 50 mL/min, pregnant or nursing women, alcohol abuse (quantified as consumption of 30 g/day), previous diagnosis of a hematological disorder, a previous episode of acute pancreatitis or known chronic pancreatitis, previous diagnosis of liver disease, and known heart failure with EF < 50%.

In the data analysis, patients were separated into two groups. The first group consisted of patients started on full-dose thiopurine defined as an AZA dose ≥ 2.0–2.5 mg/kg/d or 6-MP dose ≥ 1.0–1.5 mg/kg/d. The second group consisted of patients that were started on low-dose thiopurine, defined as an AZA dose < 2.0 mg/kg/d or 6 MP < 1.0 mg/kg/d, with slowly incremental increases. Reasons for choosing one dose over the other depended on the choice of the treating gastroenterologist at the time of initiation of medication as per routine clinical practice. Both groups were compared for safety and efficacy data. The study was approved by the MUHC's Research Ethics Committee.

Diagnosis of Crohn's disease was based on clinical, biochemical, pathological, and endoscopic findings. The Montreal classification was used to describe and collect information pertaining to disease location (ileum [L1], colon [L2], and ileocolon [L3]), behaviour (nonstricturing nonpenetrating [B1], stricturing [B2], and penetrating [B3]), and perianal disease [19]. Demographic, clinical variables, and laboratory values were collected and included. Further details such as hospitalizations, complications of underlying disease with outcomes such as perforation, obstruction, fistulizing disease, steroid use, need for surgery, and need for antibiotics were also collected.

### 2.1. Safety

Patients included in the safety analysis had to respect the previously stated inclusion and exclusion criteria but did not need to have a Harvey-Bradshaw index (HBI) documented at initiation of therapy. Frequency and timing of adverse events including gastric intolerance, myelosuppression, hepatitis, pancreatitis, and others were compared between both groups. Gastric intolerance was defined as presence of nausea and/or vomiting and/or epigastric pain attributed to thiopurine therapy. Leucopenia was defined as a WBC < 4 × 10^9^/L, neutropenia as an ANC < 1.5 × 10^9^/L, thrombocytopenia as a platelet count < 100 × 10^9^/L, anemia as hemoglobin < 100 g/L, pancreatitis as a 2-time or more elevation in lipase and amylase, and hepatitis as a 2-time or more elevation in AST, ALT, ALP, GGT, or clinical jaundice. Safety-related events were identified retrospectively through chart review and laboratory values.

### 2.2. Efficacy

Patients included in the efficacy analysis needed to have a documented HBI before initiation of therapy as determined through the chart review. Main efficacy endpoints evaluated induction of clinical remission and maintenance of remission in patients started on full-dose or low-dose thiopurine. Response to therapy was assessed at 3, 6, 9, and 12 months when documentation of clinic visits was available. Other measures of efficacy included the frequency of steroid-free remission as well as the timing and nature of treatment failure. Disease activity was assessed with the HBI. Clinical remission was defined as a HBI ≤ 4. Maintenance of clinical remission was defined as a HBI ≤ 4 at 2 consecutive visits. Steroid-free remission was achieved when patients in clinical remission were corticosteroid-free. Treatment failure was defined as discontinuation of thiopurine for persistent active CD, addition of anti-TNF-alpha medications, or CD complications including perforation, new fistulizing disease, abscess or phlegmon formation, hospitalization, and need for surgery.

### 2.3. Method of TPMT Activity Measurement

We established TPMT phenotypic activity in all patients prior to initiation of therapy. Patient's blood samples were collected in EDTA tubes and stored at 4°C and then centrifuged at 2500 rpm for 10 min. Red blood cells (RBC) were isolated and washed with physiological saline. Packed RBC were resuspended in saline and stored at −80°C. TPMT activity in RBC lysates was determined using a method previously described by Ford and Berg in 2003 with some modifications [20]. The specific activity of RBC TPMT enzyme was expressed as nmol 6-methylthioguanine/hr/g Hgb. The hemoglobin content in RBC lysates was determined using an automated hematology analyzer (Sysmex XE-5000, Sysmex Corporation, Kobe, Japan).

### 2.4. Sample Size

For our primary outcome, approximately 66 patients per group should be enrolled in the study for a total of 132. This will allow detecting a 25% higher incidence of clinical response in the full-dose group with a power of 80% and type 1 error of 0.05 for an incidence of 50% in the low-dose group [21, 22].

### 2.5. Statistical Method

For the efficacy analysis, we only included patients with HBI at initiation of therapy. For the safety analysis, we included all patients that satisfied the preset inclusion criteria. We used Fisher's exact test and Wilcoxon rank sum test to examine the significance of the association between treatment groups and patient's characteristics. To examine the efficacy of treatment, we conducted both within-group and between-group comparisons separately. The within-group comparison looked at the changes in HBI within each treatment group at each visit using paired t-test and signed-rank test, respectively. We performed a multivariate logistic regression to assess possible explanatory variables besides the initial treatment group. Adjusted odds ratios (aOR), 95% confidence intervals (CI), and *p* values were reported for the multivariate results. A similar approach was used for treatment failure and safety data. Also, we used the Kaplan-Meier plots for toxicity. *p* values of less than 0.05 were regarded as statically significant. All analyses were performed with SAS, version 9.3 (SAS Institute).

## 3. Results

### 3.1. Patient Characteristics

Our study included 134 patients, with 85 in the full-dose group and 49 in the low-dose group. 24 patients in the full-dose group and 21 in the low-dose group were excluded from the efficacy analysis as they did not have an HBI documented at initiation of therapy. The baseline characteristics of the retained patients are reported below (Table 1). Comparing both groups, patients in the full-dose group were younger (28 years versus 36 years, *p* = 0.0463), had higher TPMT values (62 nmol/hr/g Hgb versus 53 nmol/hr/g Hgb, *p* = 0.0002), had shorter disease duration (2 years versus 9 years, *p* = 0.0124), and were less likely to have perianal disease (3 (5%) versus 7 (25%), *p* = 0.0096). Importantly, the use of steroids and HBI at initiation of therapy were not different between both groups. The median weight-based dose at initiation of therapy was 1.32 mg/kg/day (Q1 = 1.06, Q3 = 1.59) in the low-dose group compared to 2.38 mg/kg/day (Q1 = 2.21, Q3 = 2.50) in the full-dose group for patients started on AZA, and 0.85 mg/kg/day (Q1 = 0.78, Q3 = 0.92) compared to 1.34 mg/kg/day (Q1 = 1.18, Q3 = 1.46) for patients started on 6 MP. Of the 49 patients in the low-dose group, 10 had a dose escalation within the first 4 weeks of therapy (median of 2 weeks). At the end of the first 4 weeks, 7 patients that were initially started on the low-dose regimen were on full-dose thiopurine and 42 were still on low dose.

### 3.2. Safety

**Figure 1:**
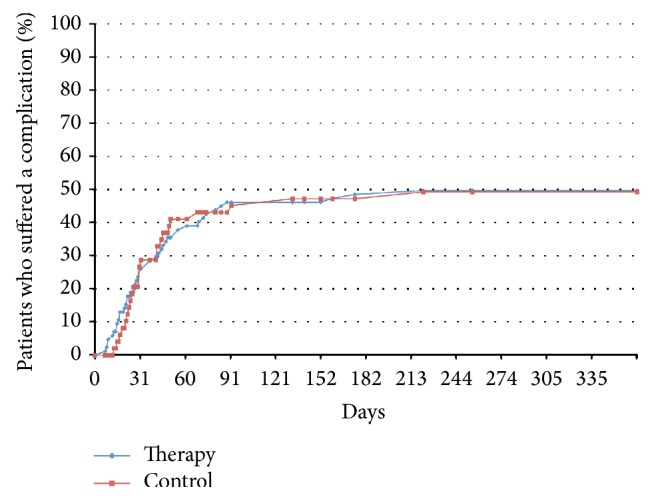
Incidence of toxicity in the full-dose (therapy) group compared to the low-dose (control) group.

A total of 64 (48%) patients had complications related to thiopurine use. There was no difference in total complications suffered by patients in the full-dose or low-dose treatment groups (42 (49%) versus 24 (49%), OR = 1.02, 95% CI 0.50–2.06, *p* = 1) (Figure 1). This remained true after adjustment of covariates (OR = 1.35, 95% CI 0.62–2.91, *p* = 0.45) and was also true when looking at individual complications (Table 2). There were similar rates of thiopurine discontinuation due to side effects: 24 (28%) patients in the full-dose group and 19 (39%) in the low-dose group (OR = 0.62, 95% CI 0.30–1.31, *p* = 0.25).

On multivariate regression analysis, TPMT level was not associated with the occurrence of complications. Older age seemed to be an independent predictor of complication (OR = 1.058, 95% CI 1.03–1.09, *p* = 0.0002) and discontinuation of medication (OR = 1.035, 95% CI 1.01–1.06, *p* = 0.0104).

Overall, two patients (1.5%) required admission to the hospital for treatment of related complications. Both were in the low-dose group. The first patient, who had a TPMT = 73 nmol/hr/g Hgb, presented at day 30 of therapy with pancreatitis. The second patient, who had a TPMT = 33 nmol/hr/g Hgb, developed leucopenia (minimum WBC = 1.63 × 10^9^/L), thrombocytopenia (minimum platelets = 33 × 10^9^/L), and anemia (minimum Hgb = 83 g/L). This was first detected 42 days after initiation of thiopurine therapy despite taking AZA at 1.3 mg/kg/d, weight-adjusted dose. Cytopenias fully recovered 1 month after cessation of AZA, and no infectious complications were reported while monitored.

### 3.3. Timing of Toxicity

At 1 month, 22 (26%) complications in the full-dose group and 14 (29%) complications in the low-dose group were attributed to thiopurine use. This represents 22 out of 42 (52%) complications and 14 out of 24 (58%) complications, respectively. At 3 months, 39 out of 42 (93%) complications and 22 out of 24 (92%) complications were noted, respectively. Overall, 64 patients suffered from complications attributed to thiopurine use with 58 patients presenting with such complaints within 3 months of therapy, accounting for 92% of cases.

### 3.4. Efficacy

Clinical response in the full-dose group at 3 months was 56% compared to 25% in the low-dose group (OR = 3.8, 95% CI 0.83–17.58, *p* = 0.0939) and 69% compared to 27% at 6 months (OR = 5.9, 95% CI 1.08–32.00, *p* = 0.0542). The differences in rates of induction of clinical remission, maintenance of remission, and steroid-free remission were not statistically significant between the full-dose group and the low-dose group (Tables 3 and 4).

### 3.5. Treatment Failure

Over the follow-up period, patients in the full-dose group had similar rates of treatment failure when compared to the group started on low-dose thiopurine (22 (26%) versus 12 (24%), OR = 1.08, 95% CI 0.48–2.43, *p* = 1). On a multivariate logistic regression model, it seemed that younger patients were more likely to experience treatment failure (OR = 0.94, 95% CI 0.90–0.98, *p* = 0.0086).

## 4. Discussion

Our study's main objective was to evaluate the safety of weight-based thiopurine dosing in a real-world setting while also monitoring efficacy. Our study demonstrated that initiating patients with normal TPMT on full-dose thiopurine was safe as it did not lead to higher rates of complications. Both treatment arms had nearly identical rates of total complications, including when analyzing each specific complication. Therefore, our results do not support starting at a lower dose and then slowly increasing. Another possibility would be to start at the highest dose, as it is safe in patients with normal TPMT, and, through close follow-up, identify patients with gastric intolerance and then try to decrease the dose or change thiopurine, while balancing efficacy. Although we had set to identify a 15% incidence of leucopenia in the full-dose group compared to 5% incidence in the low-dose group, we would have required about 153 patients per group for a power of 80% and type 1 error of 0.05 [23].

The complication rates that we report were similar to previously published studies [3, 24, 25]. Interestingly, pancreatitis occurred more frequently in the low-dose treatment group, which is consistent with the current hypothesis that thiopurine-associated pancreatitis is not related to the TPMT enzyme pathway and may be an idiosyncratic reaction [26, 27].

While TPMT concentration and treatment arm had no impact on complication rates, our covariate analysis identified age as a statistically significant predictor of adverse event and medication discontinuation. Considering that 10–15% of the cases of IBD are diagnosed above the age of 60 and that 10–30% of the IBD population is above the age of 60, it is important to know whether this population is at higher risk of thiopurine-related complications [28]. Very few studies have reported on the impact of age on the efficacy and safety profile of thiopurines, but it seems that it is generally well tolerated [28]. It has been reported that thiopurines have been associated with an increase in nonmelanoma skin cancers and non-Hodgkin lymphomas in older patients on long-term therapy [29]. A recent systematic review also showed that elderly patients were at higher risk of medication related complications compared to younger patients while on anti-TNF therapy [30]. We believe the general guiding principles of medical treatment in the elderly (i.e., “start low and go slow”) should be applied.

Furthermore, our results show that most drug-related adverse events clustered around the first 3 months of initiation of drug therapy regardless of treatment group. Based on our observations, by 3 months, 93% of complications had occurred in the full-dose group and 92% in the low-dose group. Also, we saw half of the total complications occur within the first month of therapy. This was consistent when looking at each subset of complications. Gastrointestinal intolerance tended to present within the first few days of initiation of therapy. This highlights the importance of close monitoring in the early phase of medication initiation.

An important strength of our study is its ability to be applied in a real-world setting. Most studies now being published focus on the use of metabolite levels to predict efficacy, leucopenia, and hepatotoxicity. The reality, however, is that physicians may not have access to metabolite testing or find it cumbersome, especially when considering delays in getting results. This study highlights the safety of weight-based thiopurine dosing in patients with normal TPMT without the need to resort to metabolite testing. Ensuring adequate follow-up may help early identification of patients intolerant to thiopurines.

It is important to note that, despite having a normal TPMT level, patients are still at risk for myelotoxicity, hepatitis, and pancreatitis due to mechanisms unrelated to TPMT levels. Therefore, it remains important to continue observing laboratory values on all patients started on thiopurines.

In our descriptive analysis, we found that most patients started on low-dose thiopurine actually remained on a low dose, and only a minority of patients made it to full dose before the 3-month mark. Our study also shows that patients started on full-dose thiopurine tend towards having a significantly higher chance of clinical response at 6 months compared to the low-dose group, although our analysis is limited by its small sample size. There is also a trend towards early treatment failure in the low-dose group, which is consistent with current literature that demonstrates that thiopurines take 4 to 5 weeks to achieve a steady state [3]. The aforementioned differences are probably due to the fact that it took longer for patients started on low-dose thiopurine to achieve target doses. As highlighted earlier, there are often delays in achieving optimal thiopurine weight-based dosing, which may delay efficacy without leading to improved safety [16].

Our study has several limitations, which are a result of its retrospective nature and its small sample size. One example is that the choice of the starting dose of thiopurine was based on the treating gastroenterologist's preference and the reason for the specific choice is unknown. We assume that this was not related to the disease activity because the baseline HBI in both groups was similar. In the safety analysis, adverse events may have been missed if the patient presented to another hospital with a specific complication and then did not mention it to his treating gastroenterologist but this is unlikely to happen. Furthermore, side effects may not have been accounted for if the gastroenterologist did not document them in the chart or did not perform blood tests, or again if the patient did not mention his/her symptoms. However, this likely represents very mild or spontaneously reversible toxicity and it should be noted that the prevalence of side effects in our study is reasonably similar to that of other studies.

In summary our study compared two clinical approaches to the initiation of thiopurines in CD and assessed both efficacy and safety outcomes. The study did not find any safety signals when initiating thiopurine therapy at maximal dose versus low dose in patients with normal TPMT activity and can therefore reassure clinicians that are thinking of starting such therapy. Although our efficacy analysis is limited, it appears that full-dose initiation of thiopurine medications may improve patient symptoms more rapidly and importantly and does not seem to increase the risk of side effects or complications. Patients should still be monitored closely especially during the first three months of therapy as the majority of complications appear during this time period even in the setting of a normal TPMT.

## Figures and Tables

**Table 1:** Baseline characteristics of all patients before and after exclusion due to missing HBI.

	Safety analysis			Efficacy analysis		
	Full dose (*n* = 85)	Low dose (*n* = 49)	*p* value	Full dose (*n* = 61)	Low dose (*n* = 28)	*p* value
Age, median (Q1, Q3)	33 (24, 44)	37 (27, 54)	0.130	28 (23, 41)	36 (27, 47)	0.0463
Gender, male	32 (38%)	25 (51%)	0.149	26 (43%)	12 (43%)	1.000
TPMT, median (Q1, Q3)	61 (54, 69)	58 (48, 64)	0.013	62 (55, 69)	53 (44, 61)	0.0002
Disease duration, median (Q1, Q3) years	2 (1, 12)	7 (1, 18)	0.121	2 (0, 9)	9 (1, 18.5)	0.0124
Smoking	30 (*n* = 80) (37.5%)	11 (*n* = 44) (25%)	0.169	19 (*n* = 57) (33%)	4 (*n* = 26) (15%)	0.116
Disease location			0.589			0.788
L1	34 (40%)	24 (49%)		25 (41%)	14 (50%)	
L2	15 (18%)	8 (16%)		11 (18%)	4 (14%)	
L3	36 (42%)	17 (35%)		25 (41%)	10 (36%)	
Perianal disease	8 (9%)	9 (18%)	0.178	3 (5%)	7 (25%)	0.0096
Disease behaviour			0.072			0.564
B1	57 (67%)	23 (47%)		42 (69%)	16 (57%)	
B2	14 (16.5%)	12 (24%)		9 (15%)	6 (21.5%)	
B3	14 (16.5%)	14 (29%)		10 (16%)	6 (21.5%)	
Steroid therapy	54 (64%)	31 (63%)	1.000	36 (59%)	17 (61%)	1.000
Systemic steroids	26 (31%)	16 (33%)	0.848	17 (28%)	7 (25%)	1.000
Prior surgery	24 (28%)	14 (29%)	1.000	16 (26%)	6 (21%)	0.793
HBI, median (Q1, Q3)	4 (*n* = 84)	4 (*n* = 43)	0.364	4 (3, 5)	4 (3, 6.5)	0.368
CRP, median	6.3 (*n* = 77)	6.6 (*n* = 43)	0.928	6.7 (*n* = 55)	6.5 (*n* = 26)	0.488

**Table 2:** Side effect profile of thiopurine therapy over study period.

	Full dose (*n* = 85)	Low dose (*n* = 49)	*p* value
Patients with complications	42 (49%)	24 (49%)	1.000
GI intolerance	16 (19%)	7 (14%)	0.636
Hepatitis	5 (6%)	1 (2%)	0.415
Pancreatitis	2 (2%)	5 (10%)	0.0992
Myelosuppression	8 (9%)	5 (10%)	1.000
Others	12 (14%)	6 (12%)	1.000
Discontinued treatment	24 (28%)	19 (39%)	0.250

**Table 3:** Between-group comparison of patients who achieved clinical remission.

Visit	Full dose (*n* = 61)		Low dose (*n* = 28)		*p* value
	Total number	Remission	Total number	Remission	
3 months, *n* (%)	52	11 (21%)	20	2 (10%)	0.3297
6 months, *n* (%)	35	10 (29%)	18	3 (17%)	0.5035
9 months, *n* (%)	29	10 (34%)	18	4 (22%)	0.5156
12 months, *n* (%)	23	5 (22%)	14	2 (14%)	0.6869

**Table 4:** Between-group comparison of patients who maintained remission.

Visit	Full dose (*n* = 61)		Low dose (*n* = 28)		*p* value
	Total number	Maintenance of remission	Total number	Maintenance of remission	
3 months, *n* (%)	52	27 (52%)	20	8 (40%)	0.4353
6 months, *n* (%)	35	19 (54%)	18	7 (39%)	0.3869
9 months, *n* (%)	29	13 (45%)	18	8 (44%)	1
12 months, *n* (%)	23	11 (48%)	14	7 (50%)	1
